# Altered static and dynamic regional homogeneity in basal ganglia–thalamocortical circuits and their association with neuropsychiatric manifestations in Wilson’s disease

**DOI:** 10.3389/fnins.2026.1798718

**Published:** 2026-04-20

**Authors:** Zhihua Zhou, Wenqing Xiao, Ning Yang, Weizhao Lin, Zichao Chen, Man Liang, Jiejing Li, Yunfan Wu

**Affiliations:** 1 Department of Neurology, The First Affiliated Hospital/The First Clinical Medicine School of Guangdong Pharmaceutical University, Guangzhou, China; 2 Department of Medical Imaging, The Affiliated Guangdong Second Provincial General Hospital of Jinan University, Guangzhou, China; 3 Department of Radiology, Jieyang People’s Hospital, China; 4 The Second School of Clinical Medicine, Southern Medical University, Guangdong Second Provincial General Hospital, Guangzhou, China

**Keywords:** dynamic, neuropsychiatric symptoms, regional homogeneity, static, Wilson’s disease

## Abstract

**Purpose:**

Wilson’s disease (WD) is an autosomal recessive disorder caused by ATP7B mutations, resulting in impaired copper metabolism and progressive neuropsychiatric manifestations. This study investigated spatiotemporal alterations in regional brain activity using static and dynamic resting-state fMRI with regional homogeneity (ReHo), and their relationships with clinical features.

**Methods:**

Resting-state fMRI data were acquired from WD patients and healthy controls (HCs). Static and dynamic ReHo analyses were performed to characterize local synchronization strength and temporal variability of spontaneous neural activity. Group differences were assessed across the basal ganglia, thalamus, cerebellum, and cortical regions. Associations between altered ReHo metrics and clinical measures were evaluated with FDR correction for multiple comparisons.

**Results:**

Compared with HCs, WD patients exhibited widespread ReHo abnormalities involving the basal ganglia (putamen and globus pallidus), thalamus, cerebellum, and cortical regions. Static ReHo in the left putamen and globus pallidus was positively associated with anxiety severity, while right putaminal ReHo was negatively associated with neurological severity and positively associated with disease duration. Dynamic ReHo in the left middle frontal gyrus showed negative associations with depression severity and disease duration. All brain–behavior correlations survived FDR correction, indicating robust effects.

**Conclusion:**

WD is characterized by disrupted spatiotemporal organization of local functional synchronization within cerebellar and basal ganglia–thalamo–cortical circuits. These findings support a network-level dysfunction model involving subcortical synchronization deficits and cortical temporal instability, which together underpin neuropsychiatric manifestations and disease progression.

## Introduction

Wilson’s disease (WD) is an autosomal recessive disorder caused by mutations in the ATP7B gene, which plays a critical role in copper metabolism (Dev et al., 2022). The disease is characterized by multisystem involvement, with prominent neurological manifestations including movement disorders, cognitive impairment, and psychiatric symptoms. Patients with WD exhibit a significantly increased mortality rate, approximately four times higher than that of the general population (Åberg et al., 2023). However, due to heterogeneous clinical presentations and the lack of specific early symptoms, diagnosis is often delayed until advanced stages, limiting therapeutic efficacy. As disease onset commonly occurs in childhood or adolescence, neurological and psychiatric impairments substantially affect long-term quality of life and impose a considerable socioeconomic burden. Therefore, early identification of functional brain alterations is crucial for improving clinical outcomes. However, conventional neuroimaging techniques remain limited in detecting early-stage functional abnormalities, highlighting the need for more sensitive approaches to characterize disease-related brain dysfunction.

Resting-state functional magnetic resonance imaging (rs-fMRI) has provided valuable insights into intrinsic brain activity alterations in WD. Prior studies using amplitude-based measures, such as amplitude of low-frequency fluctuations (ALFF), have demonstrated abnormal regional spontaneous activity (Hu et al., 2017). Frequency-specific ALFF analyses further suggest band-dependent functional disruptions. In addition, combined cerebral blood flow (CBF) and functional connectivity studies have revealed impaired neurovascular coupling, indicating widespread cortical and subcortical dysfunction (Hu et al., 2019). However, these approaches primarily capture signal amplitude or connectivity strength and do not directly assess local synchronization of spontaneous neural activity.

Regional homogeneity (ReHo) is a resting-state metric that reflects the temporal synchronization of a voxel with its neighboring voxels, thereby characterizing local functional coherence. Static ReHo captures average synchronization across the entire scanning period, whereas dynamic ReHo quantifies temporal variability using a sliding-window approach, enabling assessment of time-varying local brain activity. This dynamic framework provides a more sensitive perspective for detecting fluctuations in neural synchronization. ReHo has been widely applied in neuropsychiatric disorders, including Parkinson’s disease, Alzheimer’s disease, schizophrenia, and depression, demonstrating robust sensitivity to disease-related functional abnormalities. However, in WD, existing studies have predominantly focused on static ReHo alterations, particularly in subcortical regions such as the basal ganglia and thalamus. The dynamic characteristics of both subcortical and cortical networks remain largely unexplored.

To address these gaps, the present study integrates static and dynamic ReHo analyses to systematically investigate spatiotemporal alterations in local functional synchronization in WD. Furthermore, we examined the relationships between ReHo abnormalities and neuropsychiatric clinical features to improve understanding of the neural mechanisms underlying disease progression. We hypothesize that WD is associated with disrupted spatiotemporal organization of local brain activity within cortico-subcortical networks, which contributes to its neuropsychiatric manifestations.

## Material and methods

### Subjects

This study was approved by the Ethics Committee of the Guangdong Second Provincial General Hospital and was conducted in accordance with the Declaration of Helsinki. Written informed consent was obtained from all participants prior to enrollment. From 2020 to 2023, a total of 32 patients with WD and 31 age-, sex-, and education-matched healthy controls (HCs) were recruited. All participants were right-handed. WD patients were inpatients from the Department of Neurology, Guangdong Pharmaceutical University Affiliated Hospital, and were diagnosed with the neuropsychiatric subtype of WD according to the 2022 Guidelines for the Diagnosis and Treatment of Hepatolenticular Degeneration (Hou et al., 2022).

The inclusion criteria were: Participants met the following criteria: (i) age 14–60 years; (ii) ≥ 5 years of education; (iii) IQ > 80 without intellectual disability; (iv) no history of color blindness, deafness, or blindness; (v) right-handedness; (vi) no comorbid neurological or psychiatric disorders unrelated to WD; (vii) no history of substance abuse; (viii) Mini-Mental State Examination (MMSE) score > 23. Participants were excluded if they had contraindications to MRI (e.g., pacemaker, severe claustrophobia) or other major neurological disorders. All WD patients received standard anti-copper therapy (D-penicillamine, trientine, and/or zinc supplementation) and were clinically stable at the time of MRI acquisition. No participant were receiving psychotropic medication.

Clinical assessments included the Unified Wilson’s Disease Rating Scale (UWDRS), Hamilton Anxiety Rating Scale (HAMA), and Hamilton Depression Rating Scale (HAMD). MMSE and education years were recorded for all participants.

### MR data acquisition

Resting-state fMRI data were acquired on a 3.0T Philips Ingenia scanner with a 32-channel head coil. Participants lay supine with foam padding and earplugs to reduce motion and scanner noise. Functional images were obtained using an echo-planar imaging (EPI) sequence: TR = 2,000 ms, TE = 30 ms, 33 axial slices, slice thickness = 3.5 mm, no gap, FOV = 230 × 230 mm^2^, matrix = 64 × 64, 240 volumes (∼8 min). High-resolution T1-weighted images were acquired for anatomical reference (160 sagittal slices, TR = 25 ms, TE = 4.1 ms, flip angle = 30°, voxel size = 1 × 1 × 1 mm^3^). T1WI and T2-FLAIR were used to exclude structural abnormalities. All scans were reviewed independently by two experienced neuroradiologists (> 15 years experience).

### MRI data preprocessing

Data preprocessing was performed using DPABI (V6.2) and DPARSF toolbox. Steps included: (1) Removal of the first 10 volumes; (2) Slice timing correction and realignment; (3) Exclusion criteria: translation > 1.5 mm, rotation > 1.5°, or mean framewise displacement (FD) > 2 SD above group mean; (4) Coregistration to T1 images, segmentation into GM/WM/CSF using DARTEL, normalization to MNI space (3 × 3 × 3 mm^3^); (5) Regression of nuisance covariates (Friston-24 motion parameters, WM, CSF, global signal); (6) Temporal band-pass filtering (0.01–0.08 Hz). All images were visually inspected for quality assurance. To control for structural confounding, voxel-based morphometry (VBM) was performed using the DARTEL pipeline to derive gray matter volume maps.

### Computation of static and dynamic regional homogeneity

ReHo analysis was restricted to a gray matter mask (threshold = 0.2 derived from averaged GM maps). Static ReHo (sReHo) was calculated using Kendall’s coefficient of concordance (KCC) among a 27-voxel neighborhood (Zang et al., 2004). Individual maps were standardized to z-scores. Dynamic ReHo (dReHo) was computed using a sliding-window approach (window size = 30 TRs; step = 1 TR). ReHo maps were generated for each window, and voxel-wise temporal variability was estimated. Final maps were standardized using z-transformation. All maps were spatially smoothed using an 8-mm FWHM Gaussian kernel prior to statistical analysis.

### Statistical analysis

Demographic and clinical variables were analyzed using SPSS 20.0. Normality was assessed using the Shapiro–Wilk test. Group comparisons were performed using two-sample *t*-tests (*p* < 0.05). Voxel-wise comparisons of sReHo and dReHo were conducted using a general linear model (GLM) in DPABI/SPM12. Age, sex, education, and mean FD were included as covariates. Multiple comparisons were corrected using Gaussian Random Field (GRF) theory (voxel-level *p* < 0.005, cluster-level *p* < 0.05, two-tailed). Effect sizes (Cohen’s *d*) were calculated for significant clusters.

### Structural validation (VBM-controlled ANCOVA)

To ensure that functional alterations were independent of gray matter atrophy, mean GMV from each significant cluster was extracted and included as an additional covariate in a secondary ANCOVA model. Only clusters surviving this correction were retained for further analysis.

### Correlation analysis

Pearson correlation analyses were conducted within the WD group. Age and sex were included as covariates. To control for multiple comparisons across six correlation analyses, False Discovery Rate (FDR) correction was applied, with a corrected threshold of *q* < 0.05. Correlations that did not survive FDR correction were explicitly labeled as exploratory and interpreted with caution.

### *Post hoc* power analysis

*Post hoc* power analysis was performed using G*Power 3.1 based on observed effect sizes (Cohen’s *d* range: 1.09–1.70) and sample size (32 WD, 31 HC). The analysis indicated statistical power > 0.90 for detecting large effect sizes (α = 0.05, two-tailed) were retained for further analysis.

## Results

### Demographic and clinical features

A total of two WD patients were excluded due to excessive head motion based on predefined criteria. The final analysis included 32 WD patients and 31 healthy controls (HCs). No significant between-group difference in mean framewise displacement was observed (*p* > 0.05).

As summarized in Table 1, no significant group differences were found in age, sex distribution (χ^2^ test), years of education, or MMSE scores (all *p* > 0.05). In contrast, WD patients exhibited significantly higher HAMA and HAMD scores than HCs (both *p* < 0.05), indicating increased anxiety and depressive symptoms in the WD group.

**TABLE 1:** Baseline characteristics of WD patients and HC subjects.

Characteristic	WD Patients (*n* = 32)	HC participants (*n* = 31)	*P*-value
Age (years, mean ± SD)	33.50 ± 10.21	29.13 ± 7.38	0.145
Sex (M/F, n)	18/14	17/14	0.533
Disease duration (years)	13.34 ± 6.15	–	–
Education (years, mean ± SD)	13.00 ± 6.00	15.00 ± 5.00	0.150
MMSE score	29.03 ± 0.88	29.22 ± 1.76	0.592
UWDRS-N score	31.59 ± 11.16	–	–
UWDRS-P score	8.47 ± 5.28	–	–
HAMA score	24.56 ± 4.25	3.04 ± 2.44	< 0.001

Data are mean ± standard deviation; Wilson’s Disease; HC, healthy controls; HAMA, the Hamilton rating scales of anxiety; HAMD, the Hamilton rating scales of depression; UDWRS-N, neurological subscale of the Unified Wilson’s Disease Rating Scale; UDWRS-P, psychiatric subscale of the Unified Wilson’s Disease Rating Scale.

### Group differences in sReHo

**FIGURE 1:**
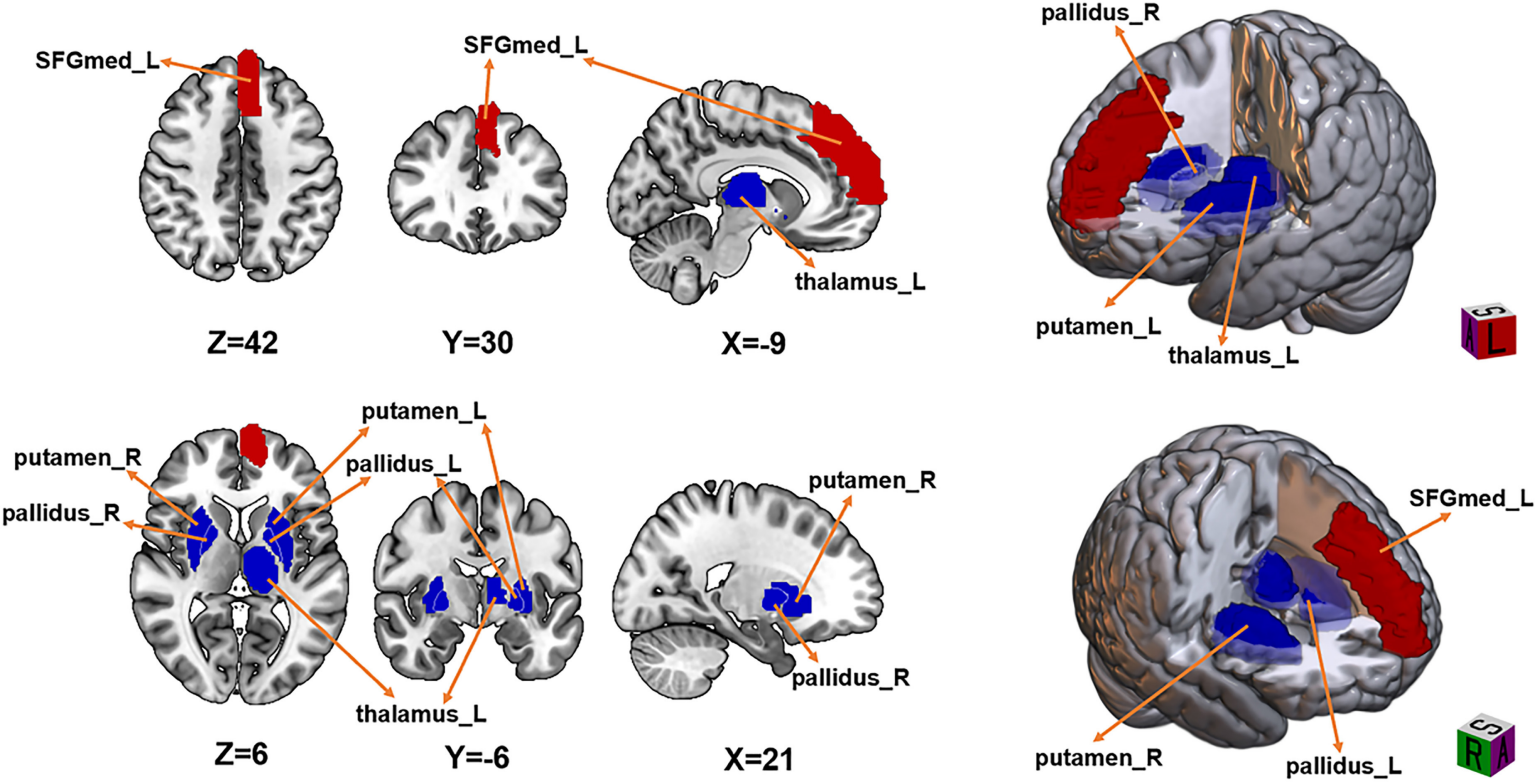
Brain regions showing significant differences in static ReHo (sReHo) between Wilson’s disease (WD) patients and healthy controls (HC). Regions in red indicate significantly increased sReHo in WD compared with HC, whereas regions in blue indicate significantly decreased sReHo in WD. The results were corrected for multiple comparisons using Gaussian Random Field (GRF) correction at the voxel level (two-tailed voxel-level threshold: *p* < 0.005, cluster-level threshold: *p* < 0.05), with a minimum cluster size > 20 voxels.

Voxel-wise comparisons were performed using Gaussian Random Field (GRF) correction (voxel-level *p* < 0.005, cluster-level *p* < 0.05, two-tailed). Compared with HCs, WD patients showed significantly increased sReHo in the left medial superior frontal gyrus (SFGmed) (*t* = 5.826, *d* = 1.49). Significantly decreased sReHo was observed in the left thalamus (*t* = −5.572, *d* = 1.43), bilateral putamen (left: *t* = −6.222, *d* = 1.59; right: *t* = −5.875, *d* = 1.50), and bilateral globus pallidus (left: *t* = −6.222, *d* = 1.59; right: *t* = −5.296, *d* = 1.36) (Figure 1 and Table 2).

**TABLE 2:** Brain regions showing significant group differences in static and dynamic ReHo (WD vs. HC).

Indices	Brain region	MNI coordinate (X,Y,Z)	Cluster Size (Voxels)	Cohen’s *d*	Peak t value
sReHo	Putamen_R	21, −6, −3	32	−1.50	−5.875
	Putamen_L	−21, 3, 30	59	−1.59	−6.222
	Globus pallidus_R	23, −8, −1	25	−1.36	−5.296
	Globus pallidus_L	−21, 10, 10	160	−1.59	−6.222
	Thalamus_L	6, −18, 6	38	−1.43	−5.572
	SFGmed_L	−9, 21, 42	150	1.49	5.826
dReHo	Globus pallidus_L	−18, 4, −6	26	−1.38	−5.390
	ANG_R	45, −51, 24	76	1.29	5.034
	SFGmed_L	−9, 39, 21	105	1.37	5.335
	Cerebellum_Crus1_R	30, −96, −18	31	1.09	4.270
	PCUN_L	−18, −60, 30	127	1.70	6.643
	MFG_R	30, 48, 3	38	1.38	5.375
	MFG_L	−27, 51, 3	31	1.23	4.818
	IOG_R	42, −90, −9	44	1.39	5.449

All group comparisons were corrected for multiple comparisons using Gaussian Random Field (GRF) theory (voxel-level *p* < 0.005, cluster-level *p* < 0.05, two-tailed). Effect sizes (Cohen’s *d*) were calculated based on peak t statistics from independent-sample *t*-tests (WD: *n* = 32; HC: *n* = 31). No additional FDR correction was applied, as GRF correction was used for voxel-wise imaging analyses.

### Group differences in dReHo

Relative to HCs, WD patients exhibited increased dReHo in the left SFGmed (*t* = 5.335, *d* = 1.37), left precuneus (*t* = 6.643, *d* = 1.70), right cerebellum Crus I (*t* = 4.270, *d* = 1.09), right angular gyrus (*t* = 5.034, *d* = 1.29), right inferior occipital gyrus (*t* = 5.449, *d* = 1.39), and bilateral middle frontal gyrus (left: *t* = 4.818, *d* = 1.23; right: *t* = 5.375, *d* = 1.38).

**FIGURE 2:**
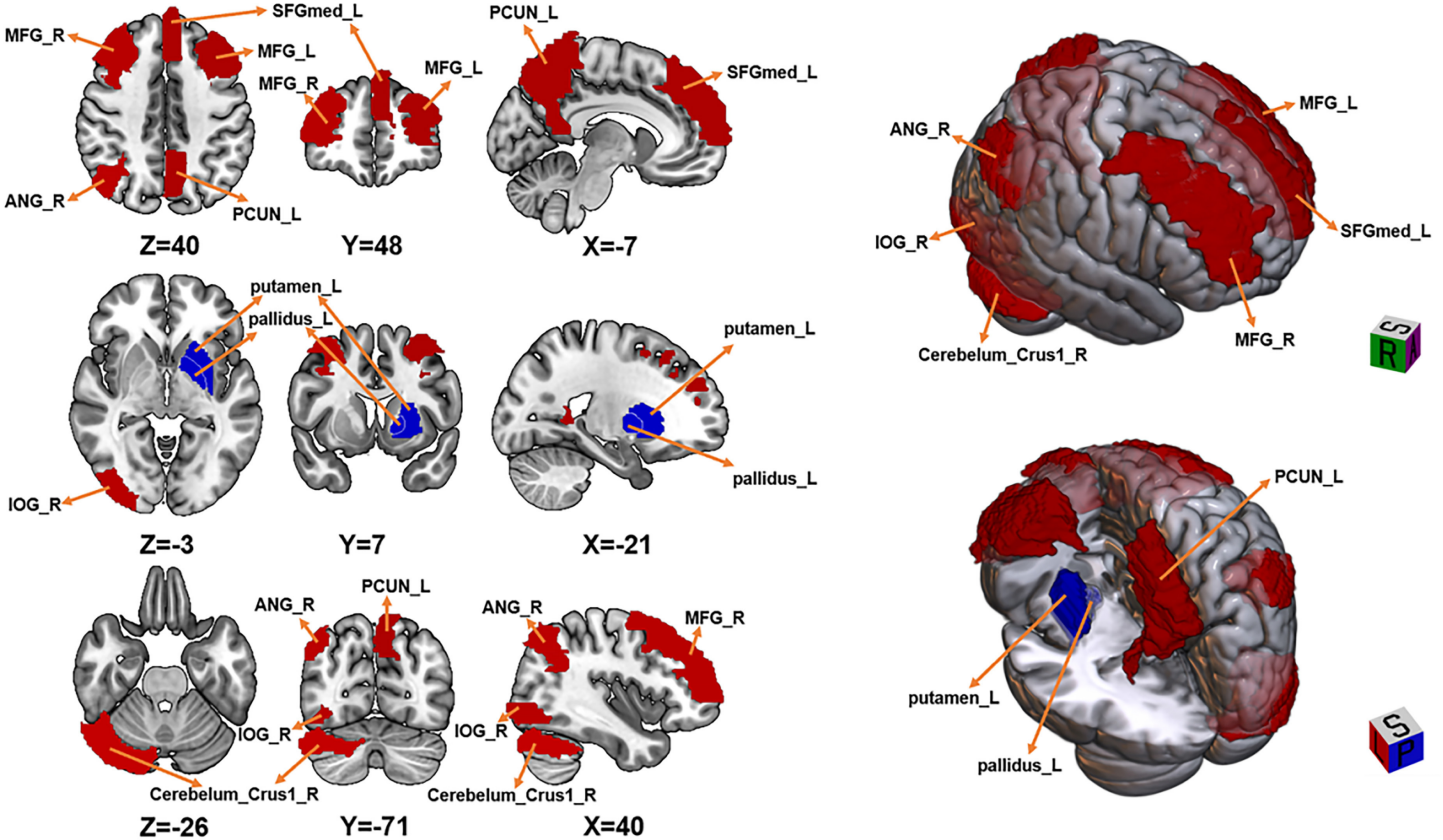
Brain regions showing significant differences in dynamic ReHo (dReHo) between Wilson’s disease (WD) patients and healthy controls (HC). Regions in red indicate significantly increased dReHo in WD compared with HC, whereas regions in blue indicate significantly decreased dReHo in WD compared with HC. The results were corrected for multiple comparisons using Gaussian Random Field (GRF) theory at the voxel level (two-tailed voxel-level threshold: *p* < 0.005, cluster-level threshold: *p* < 0.05), with a minimum cluster size > 20 voxels.

Decreased dReHo was identified in the left putamen (*t* = −6.222, *d* = 1.59) and left globus pallidus (*t* = −5.390, *d* = 1.38) (Figure 2 and Table 2).

### Correlation analysis

**FIGURE 3:**
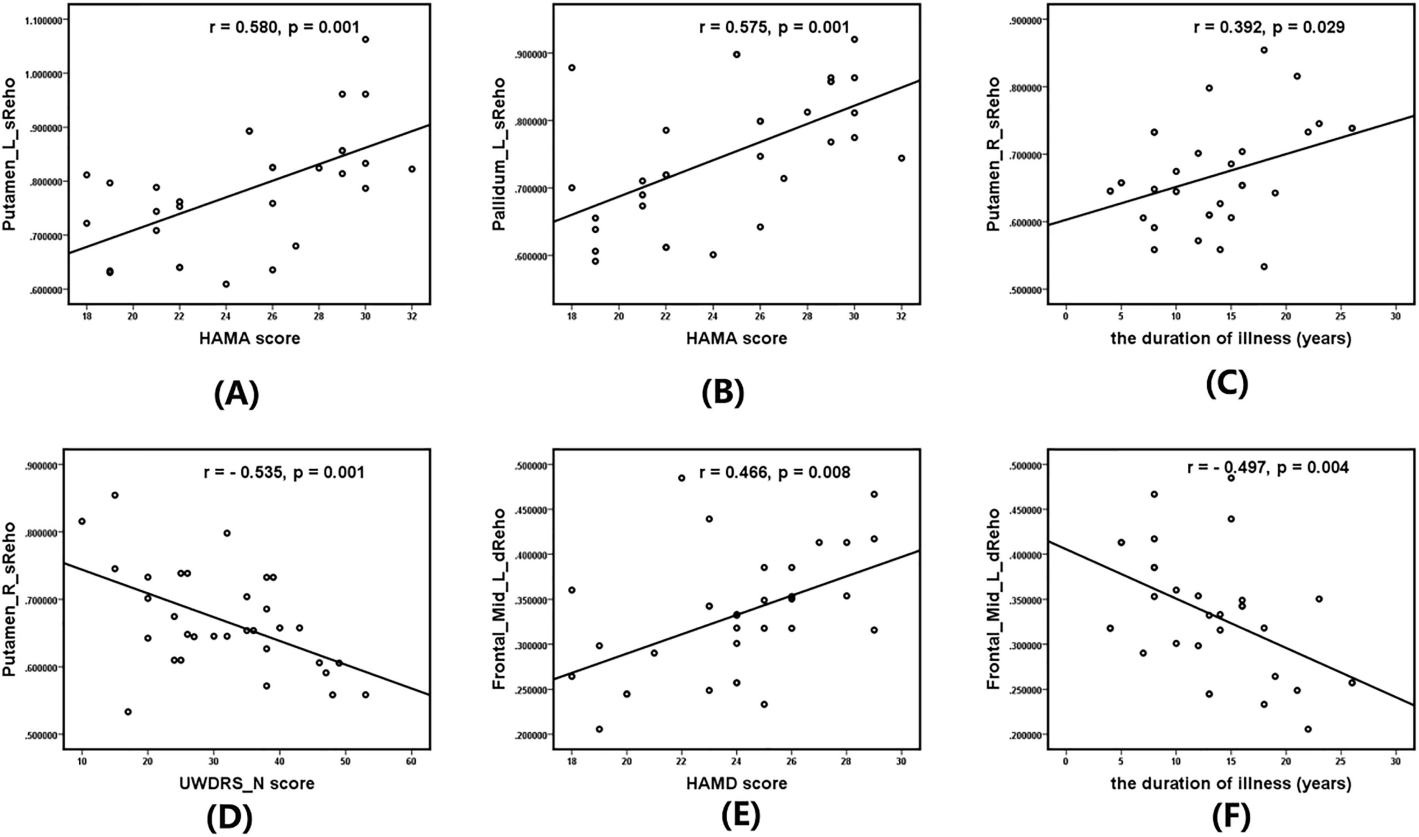
Significant correlations between sReHo/dReHo values and neurocognitive, emotional scale scores, and disease duration in patients with Wilson’s disease (WD). **(A)** Correlation between sReHo in the left putamen and HAMA scores. **(B)** Correlation between sReHo in the left globus pallidus and HAMA scores. **(C)** Correlation between sReHo in the right putamen and Unified Wilson’s Disease Rating Scale–neurological examination (UWDRS-N) scores. **(D)** Correlation between sReHo in the right putamen and disease duration. **(E)** Correlation between dReHo in the left middle frontal gyrus (MFG) and HAMD scores. **(F)** Correlation between dReHo in the left middle frontal gyrus (MFG) and disease duration.

Within the WD group (*n* = 32), Pearson correlation analyses were performed, and 95% confidence intervals were estimated using Fisher’s z transformation. Significant positive correlations were observed between sReHo in the left putamen and HAMA scores (*r* = 0.580, 95% CI [0.28, 0.77], *p* = 0.001), and between sReHo in the left globus pallidus and HAMA scores (*r* = 0.575, 95% CI [0.27, 0.76], *p* = 0.001). In addition, sReHo in the right putamen was negatively correlated with UWDRS-N scores (*r* = −0.535, 95% CI [−0.74, −0.22], *p* = 0.002) and positively correlated with disease duration (*r* = 0.392, 95% CI [0.04, 0.66], *p* = 0.029). dReHo in the left MFG was negatively correlated with both HAMD scores (*r* = −0.466, 95% CI [−0.69, −0.13], *p* = 0.008) and disease duration (*r* = −0.497, 95% CI [−0.71, −0.17], *p* = 0.004) (Figure 3). All reported correlations remained significant after false discovery rate (FDR) correction (*q* < 0.05), indicating robustness against multiple comparison bias.

### *Post hoc* power analysis

A *post hoc* power analysis was conducted using G*Power 3.1 based on observed effect sizes (Cohen’s *d* = 1.09–1.70) and the current sample size (32 WD patients and 31 HCs). The results indicated that statistical power exceeded 0.90 (α = 0.05, two-tailed) for detecting large effects, suggesting that the study was adequately powered to detect the observed group differences.

## Discussion

In this study, we integrated static and dynamic ReHo analyses to characterize spatiotemporal alterations of local spontaneous brain activity in WD. Compared with healthy controls, WD patients showed widespread ReHo abnormalities involving the basal ganglia, thalamus, cerebellum, and distributed cortical regions, including the prefrontal, parietal, and occipital cortices. Importantly, these alterations were significantly associated with anxiety, depression, neurological severity, and disease duration, indicating a robust coupling between local functional synchronization and clinical manifestations.

ReHo reflects local temporal synchronization of spontaneous neural activity and is closely related to neuronal coherence and neurovascular coupling underlying the BOLD signal. Increased ReHo may indicate aberrant hyper-synchronization or compensatory recruitment, whereas decreased ReHo reflects disrupted local functional integration. Notably, all brain–behavior associations survived FDR correction, supporting the robustness and clinical relevance of these findings.

A key finding is the consistent involvement of the basal ganglia–thalamocortical system. Both static and dynamic ReHo abnormalities were observed in the putamen and globus pallidus, together with reduced static ReHo in the thalamus. These regions form a core circuit supporting motor control, emotion regulation, and executive function, while the thalamus acts as a central integrative hub for distributed brain networks (Alexander and Crutcher, 1990; Wichmann and DeLong, 2016; Shine et al., 2023). Consistent with prior structural and pathological evidence of basal ganglia and thalamic vulnerability in WD (Starosta-Rubinstein et al., 1987; King et al., 1996), our findings further demonstrate that functional disruption of these hubs is closely linked to clinical severity. The observed correlations between putaminal ReHo and neurological impairment and disease duration suggest a progression-related shift from functional disruption toward maladaptive or compensatory reorganization, although causal inference is limited by the cross-sectional design.

A major contribution of this study is the dissociation between static and dynamic ReHo. Static ReHo primarily captured persistent synchronization deficits in subcortical hubs, whereas dynamic ReHo showed limited overlap in these regions. This divergence suggests that WD-related dysfunction extends beyond reduced baseline synchronization to include altered temporal stability of neural activity. Together, these findings highlight a spatiotemporal breakdown of intrinsic brain organization, emphasizing that WD is not only characterized by reduced activity coherence but also by instability of neural dynamics.

Beyond subcortical regions, dynamic ReHo revealed widespread cortical involvement across prefrontal, parietal, and occipital association areas, including the medial and middle frontal gyri, angular gyrus, precuneus, and inferior occipital gyrus. These regions are central to executive control, attention, memory, language, and visuospatial processing (Cavanna and Trimble, 2006; Lau et al., 2019). The observed abnormalities indicate that WD is not confined to basal ganglia pathology but represents a distributed cortical–subcortical network disorder. Increased dynamic variability may reflect instability of local neural assemblies, whereas decreased ReHo may indicate impaired functional integration. The coexistence of both patterns suggests concurrent neurodegenerative disruption and compensatory network reorganization.

Dynamic abnormalities in the cerebellum further extend WD pathology to motor coordination systems. Clinically, cerebellar ataxia is a hallmark feature of WD, including gait disturbance, tremor, and coordination deficits (Dusek et al., 2019). Neuropathological evidence of Purkinje cell loss and cerebellar atrophy (Smeets and Verbeek, 2014) supports our finding that cerebellar network instability contributes to motor dysfunction at the systems level.

Importantly, clinical correlation analyses demonstrated that increased basal ganglia ReHo was associated with higher anxiety, whereas decreased medial frontal ReHo was associated with more severe depression and longer disease duration. These results implicate basal ganglia–thalamo–cortical circuits in the neuropsychiatric manifestations of WD and suggest that progressive disease burden contributes to cumulative disruption of emotional and cognitive regulation systems.

Although previous static ReHo studies have reported similar subcortical abnormalities (Zhou et al., 2018), the present study extends these findings by integrating static and dynamic ReHo within a unified spatiotemporal framework. This approach reveals complementary but partially dissociable patterns of brain dysfunction, demonstrating that WD involves not only reduced subcortical synchronization but also instability of cortical network dynamics. These results support the interpretation of WD as a distributed network-level disorder rather than a focal basal ganglia disease.

Several limitations should be acknowledged. First, despite GRF correction, the relatively small sample size and cross-sectional design limit statistical power and causal inference. Second, dynamic ReHo estimation is sensitive to sliding-window parameters, and its robustness across parameter settings requires further validation. Third, susceptibility effects due to copper and iron deposition in deep gray matter may influence BOLD signals. As quantitative susceptibility mapping, SWI, and systematic tSNR evaluation were not available, residual signal bias cannot be excluded. In addition, treatment status was not controlled. Although we controlled for gray matter volume at the cluster level, voxel-wise structural–functional coupling was not fully accounted for. Future multimodal studies integrating structural, diffusion, and susceptibility imaging are needed to validate and extend these findings.

In conclusion, WD is characterized by widespread spatiotemporal abnormalities of local neural synchronization across both subcortical and cortical networks. These alterations reflect a breakdown of intrinsic brain organization involving both reduced synchronization strength and impaired temporal stability. Together, they provide a network-level framework for understanding motor, cognitive, and emotional impairments in WD and support the view that WD is a distributed spatiotemporal brain network disorder.

## Conclusion

By integrating static and dynamic ReHo analyses, this study suggests that abnormal local neural synchronization within cerebellar and basal ganglia–thalamocortical circuits is associated with neuropsychiatric manifestations in WD. These findings indicate that decreased or increased ReHo may reflect disrupted and compensatory neural processes, respectively. Together with clinical correlations, the results support a spatiotemporal network imbalance underlying neuropsychiatric symptoms in WD.

## Data Availability

The original contributions presented in the study are included in the article/supplementary material, further inquiries can be directed to the corresponding author.
